# Fragment‐based drug discovery—the importance of high‐quality molecule libraries

**DOI:** 10.1002/1878-0261.13277

**Published:** 2022-07-10

**Authors:** Marta Bon, Alan Bilsland, Justin Bower, Kirsten McAulay

**Affiliations:** ^1^ Cancer Research Horizons Cancer Research UK Beatson Institute Glasgow UK

**Keywords:** covalent fragments, fraglites, fragment library, fragment‐based drug discovery, machine learning, virtual screening

## Abstract

Fragment‐based drug discovery (FBDD) is now established as a complementary approach to high‐throughput screening (HTS). Contrary to HTS, where large libraries of drug‐like molecules are screened, FBDD screens involve smaller and less complex molecules which, despite a low affinity to protein targets, display more ‘atom‐efficient’ binding interactions than larger molecules. Fragment hits can, therefore, serve as a more efficient start point for subsequent optimisation, particularly for hard‐to‐drug targets. Since the number of possible molecules increases exponentially with molecular size, small fragment libraries allow for a proportionately greater coverage of their respective ‘chemical space’ compared with larger HTS libraries comprising larger molecules. However, good library design is essential to ensure optimal chemical and pharmacophore diversity, molecular complexity, and physicochemical characteristics. In this review, we describe our views on fragment library design, and on what constitutes a good fragment from a medicinal and computational chemistry perspective. We highlight emerging chemical and computational technologies in FBDD and discuss strategies for optimising fragment hits. The impact of novel FBDD approaches is already being felt, with the recent approval of the covalent KRAS^G12C^ inhibitor sotorasib highlighting the utility of FBDD against targets that were long considered undruggable.

## Graphical abstract

Fragment‐based drug discovery (FBDD) uses collections (libraries) of smaller, less complex molecules (fragments) than drug‐like molecules used in high‐throughput screening. Herein, we highlight the advantages of FBDD, which has already resulted in several marketed drugs. Finding quality FBDD hits depends on good library design. We describe our views on chemical and computational aspects of library design and discuss emerging technologies.

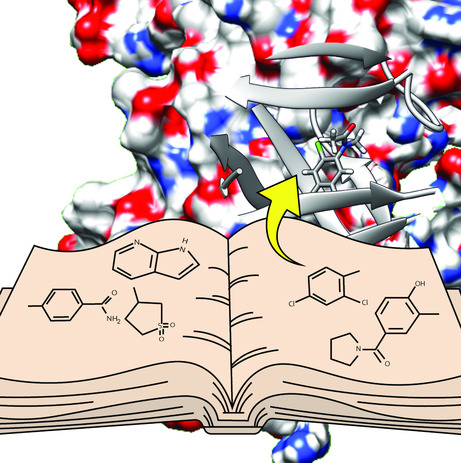

**Abbreviations**
2D/3D: 2 dimensional/3 dimensional
5‐HT1A: 5‐hydroxytryptamine receptor 1A
5‐HT2A: 5‐hydroxytryptamine receptor 2A
ADME/ADMET: absorption, distribution, metabolism, excretion and toxicity
AE: autoencoder
AI: artificial intelligence
BACE1: β‐site amyloid precursor protein cleaving enzyme 1
BRD4: bromodomain‐containing protein 4
BRICS: breaking of retrosynthetically interesting chemical substructures
CDK2: cyclin‐dependent kinase 2
cLogD: calculated logarithm of distribution coefficient
cLogP: calculated logarithm of distribution coefficient
DRD2: dopamine receptor D2
EGFR: epidermal growth factor receptor
FBDD: fragment‐based drug discovery
FDA: United States Food and Drug Administration
FEP: free energy perturbation
Fsp3: fraction of sp3‐hybridised carbon atoms
GAN: generative adversarial network
GPCR: G‐protein coupled receptor
HAC: heavy atom count
HBA: hydrogen bond acceptor count
HBD: hydrogen bond donor count
HTS: high‐throughput screening
JAK: Janus kinase
*k*_d_
: dissociation constant
*k*_i_
: inhibition constant
*k*_inact_
: inactivation rate constant
KRAS: Kirsten rat sarcoma virus oncogene homologue
LC–MS/MS: liquid chromatography with tandem mass spectrometry
LogSw: logarithm of water solubility (calculated)
MD: molecular dynamics
MM/PBSA: molecular mechanics Poisson–Boltzmann surface area
MS: mass spectrometry
NMR: nuclear magnetic resonance
PAINS: pan‐assay interference compounds
PBF: plane of best fit
PMI: principal moments of inertia
PPI: protein–protein interaction
PSA: polar surface area
QSAR: quantitative structure–activity relationship (model)
QSPR: quantitative structure–property relationship (model)
RECAP: retrosynthetic combinatorial analysis procedure
RL: reinforcement learning
RNN: recurrent neural network
Ro3: rule of three
SAR: structure–activity relationship
SARS‐CoV‐2: severe acute respiratory syndrome coronavirus 2
SELFIES: self‐referencing embedded strings
SETD2: Su(var)3‐9, enhancer‐of‐zeste and trithorax‐domain containing 2
SMARTS: SMILES arbitrary target specification
SMILES: simplified molecular‐input line‐entry system
sp2: sp2‐hybridised atomic orbital
sp3: sp3‐hybridised atomic orbital
SPR: surface plasmon resonance
TPSA NOPS: total polar surface area including N, O, P and S atoms
VAE: variational autoencoder

## 1 Introduction

**Fig. 1:**
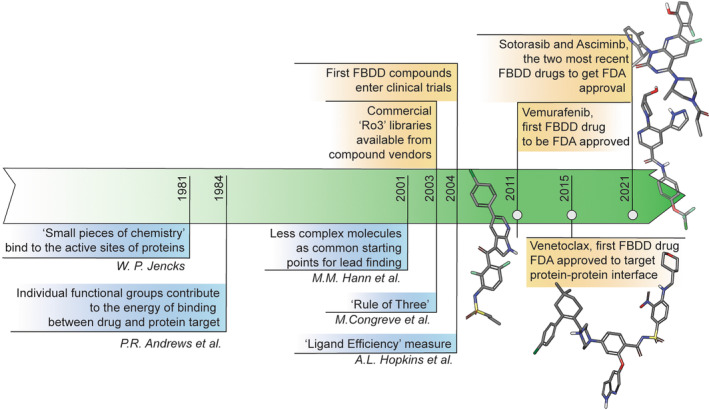
Timeline highlighting key papers influencing the course of FBDD (blue) and important dates showing its success (orange). In an early conceptual paper, Jencks outlined the additivity of binding energies for fragments of larger molecules [16]. Andrews et al. [17] subsequently estimated intrinsic binding energy contributions to ligand–receptor interactions for a range of functional groups. Based on a simple model of complementary ligand–receptor features, Hann et al. [18] proposed that molecules of lower complexity are likely to provide better starting points for drug discovery and discussed the need for highly sensitive assays. With increasing interest in fragment‐based drug discovery, commonly used metrics including ‘rule of three’ and ligand efficiency were developed [11, 19]. FBDD, fragment‐based drug discovery; FDA, United States Food and Drug Administration. [Colour figure can be viewed at wileyonlinelibrary.com]

Over the last two decades, fragment‐based drug discovery (FBDD) has proven its utility as a complementary, and highly successful, approach to high‐throughput screening (HTS) for the identification of molecules for hit to lead campaigns during which properties and potency of screening actives are extensively optimised [1, 2] (Fig. 1). To date, use of an FBDD approach has resulted in six marketed drugs, pexidartinib [3], vemurafenib [4], erdafitinib [5], venetoclax [6], sotorasib [7] and asciminib [8], as well as numerous clinical candidates.

The method has become widely used in pharma, biotech and academic institutions across the globe, with 20 fragment to lead publications reported in 2019, and 21 publications in 2020 [9, 10]. Fragment libraries are able to sample much greater chemical space than HTS libraries, with a much smaller number of compounds. Complex molecules have a greater chance of forming sub‐optimal interactions and/or clashes with the desired target, unlike fragments which are more likely to make atom‐efficient binding interactions [11, 12]. Thus, a library of only one to two thousand small molecules can easily provide quality hits for a drug discovery programme [13]. Moreover, fragment hit rates can be used as an assessment of the potential druggability of a target [14] and can be used to identify difficult‐to‐target binding regions, such as allosteric sites or small ‘hot spot’ binding pockets which are often implicated in protein–protein interactions [15]. This utility is highlighted by the success of venetoclax, one of the first drugs to target a protein–protein interaction (PPI) interface, and more recently sotorasib, which targets the KRAS G12C mutant, previously considered undruggable.

**Fig. 2:**
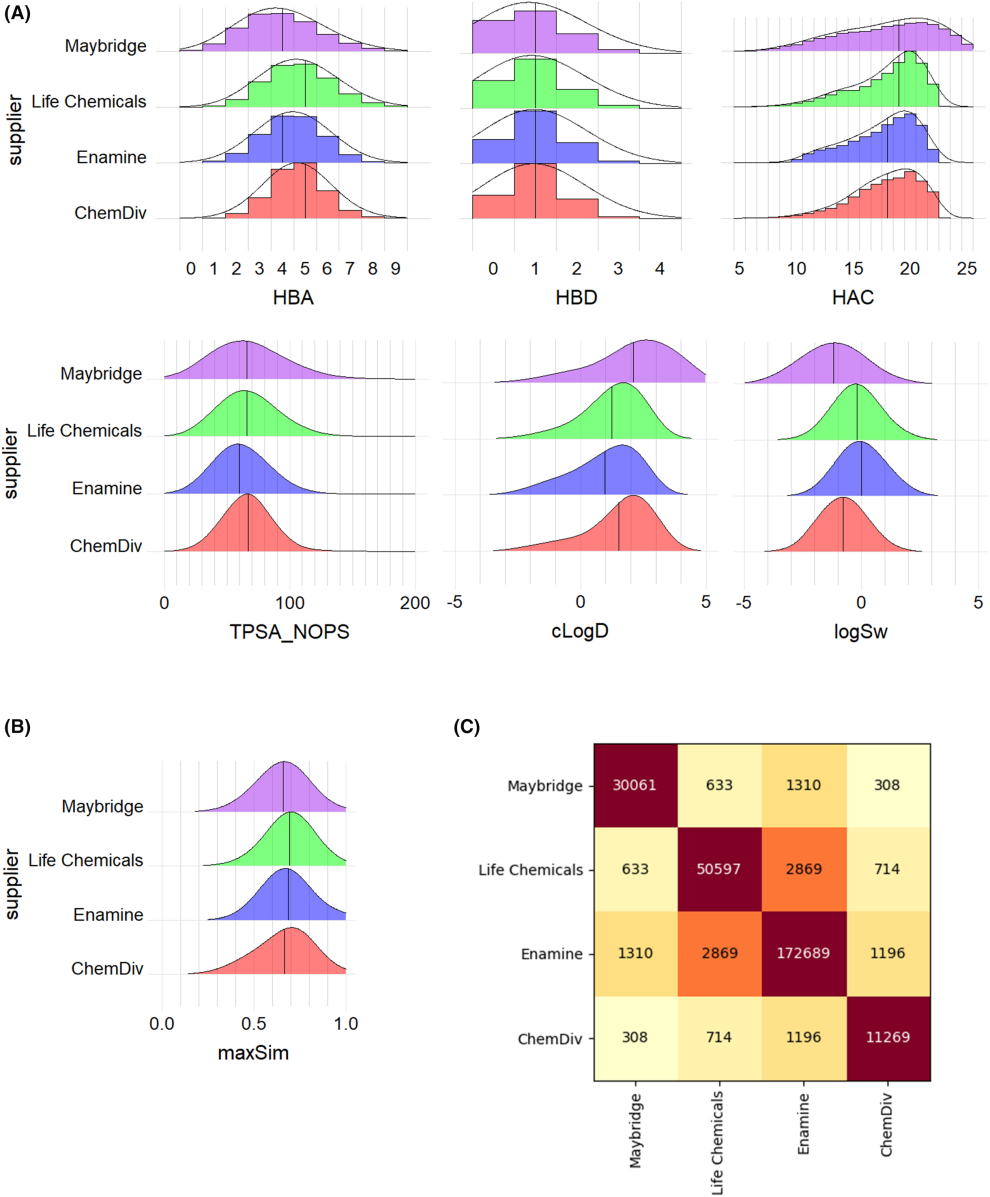
Property distributions in selected unfiltered large commercial fragment sets. (A) General fragment sets were obtained from Maybridge (30 061 compounds), Life Chemicals (50 597 compounds), Enamine (172 689 compounds) and ChemDiv (11 269 compounds). Hydrogen bond donors/acceptors, heavy atom count and total polar surface area including N, O, P and S were calculated in vortex software (Dotmatics, Bishops Stortford, UK). Predicted logD and water solubility were calculated in admet predictor software (Simulations Plus, Lancaster, CA, USA). Black lines denote mean or median for continuous or discrete properties, respectively. (B) Distributions of maximum internal similarity in the same fragment sets. For each compound, pairwise Tanimoto similarity was calculated in RDKit (RDKit: Open‐source cheminformatics; http://www.rdkit.org) against all other compounds in the set using Morgan fingerprints, radius 2. See the Section 2.2.2 for an explanation of Tanimoto similarity. For each compound, the maximum value of similarity against any other compound was retained. (C) Number of identical compounds in the same libraries. For example, 633 compounds are present in both Maybridge and Life Chemicals collections. HBA, hydrogen bond acceptor count; HBD, hydrogen bond donor count; HAC, heavy atom count; TPSA_NOPS, total polar surface area including N, O, P and S atoms; cLogD, calculated logarithm of distribution coefficient; LogSw, (calculated) logarithm of water solubility; maxSim, maximum internal similarity (as defined above). [Colour figure can be viewed at wileyonlinelibrary.com]

What defines a fragment? The accepted core definition describes a fragment as a small organic molecule, generally with ≤ 20 heavy atoms. Past fragment library design tended to focus on physicochemical properties broadly following the ‘rule of three’ (Ro3), which has become synonymous to Lipinski's rules in the fragment world [19]. These are: molecular weight ≤ 300 Da, hydrogen bond donors (HBD) ≤ 3, hydrogen bond acceptors (HBA) ≤ 3 and computed logarithm of the partition or distribution coefficient (cLogP/cLogD) ≤ 3. In addition, freely rotatable bonds ≤ 3 and polar surface area (PSA) ≤ 60 are often considered Ro3 criteria. Yet, this is not a ‘hard and fast’ set of rules and selection criteria have evolved over time. Successful fragments will often violate at least one of these rules [20], most commonly having a higher HBA count (Fig. 2).

Fragment hits tend to have weak affinities, with dissociation constant (*k*_d_) values in the μm–mm range, compared with HTS hits which generally have stronger affinities within the nm–low μm range. Thus, they often require more extensive chemistry efforts to reach a lead‐like compound, which can be particularly difficult in an academic setting. Their weaker affinities also mean that biochemical assays, which are typically used for HTS screens cannot be used as an accurate measure of fragment binding. Instead, biophysical techniques such as nuclear magnetic resonance (NMR), surface plasmon resonance (SPR), X‐Ray crystallography and thermal shift assays are typically used to probe binding, with two orthogonal methods often used to validate any hits.

Finding quality hits is largely a result of good library design; screening simple, highly attractive molecules which span a breadth of chemical space. Herein, we describe our views on fragment library design and what constitutes a good fragment.

## 2 The requirements of a fragment library

### 2.1 Currently available fragment libraries and their limitations

Fragment libraries are constructed to explore a broad range of chemical space while screening a limited number of compounds. Therefore, diversity is generally the main driver in library design. However, in some cases, it may also be beneficial to consider the target class, for example, whether specific ligand moieties known to bind to functionally related protein targets should be included. A number of fragment libraries are now commercially available, spanning a range of properties and chemical space. These are an incredibly useful starting point for library development, having generally been filtered to contain desired pharmacophore, chemical and shape diversity.

Despite this, there are some limitations to only utilising one commercially available library. The size and diversity of each library varies (Fig. 2) and so may not be optimal when compared to designing a bespoke set. Commercial libraries are also generally larger than the number of fragments required to run a successful hit identification campaign, and so each library will often need to be filtered to give a reasonable set size. While there is some overlap between commercially available compounds, normally there is a high degree of unique chemical entities contained within each set. It can, therefore, be beneficial to ‘mix and match’ to obtain desired properties and optimal diversity. Moreover, solubility [21, 22] and stability of purchasable fragments may need to be examined depending on the screening method. Low solubility can be a particular issue during FBDD, and so some vendors have now sought to provide specific ‘high solubility’ sets. Conventional organic fragment sets also tend to have a high degree of planarity, which can contribute to solubility issues, with sp2‐rich aromatic rings appearing as substructures in many compounds [23]. This partially leads back to the traditional targets which fragments have been screened against (such as kinases) and to the rise in the use of catalytic sp2–sp2 coupling reactions. Again, vendors have moved to address this by offering libraries exhibiting greater sp3 and 3D character. Regardless of the large number of commercially available fragments, it is important to try and supplement any library with noncommercially available fragments from the likes of in‐house chemistry efforts. Such scaffolds can provide a good base for future optimisation strategies.

### 2.2 How do you design a library?

#### 2.2.1 Medicinal chemistry considerations

Design and growth of a fragment set usually begins by examining and filtering commercially available collections to exclude compounds containing known toxic structures (toxicophores) and maintain desired pharmacokinetic properties (Table 1). Although these properties broadly follow the Ro3, there are several other selection criteria, which should be carefully considered. Synthetically accessible modification points on the core are important to enable growth vectors for lead optimisation. Solubility and hydrophobicity are also key factors, which can affect unwanted potential aggregation. Inclusion of HBA, HBD and other binding motifs is not only crucial to aid enthalpy‐driven binding interactions but also to ensure cLogD is within a desired range. Each fragment should be of minimal size and complexity to drive efficient interactions and avoid clashes with the target. As such, molecules with a high degree of flexibility may result in lower affinity hits due to entropic costs. Nevertheless, a balance must be struck on the inclusion of polar functionality and desirable pharmacophores, so that complexity and diversity of the set can be maintained.

**Table 1:** Typical property ranges used by the Beatson Drug Discovery Unit in filtering commercial sets, together with descriptive statistics illustrating composition of our 1H fragment set in respect of each property. All Beatson library properties calculated in admet predictor; Simulations Plus).

Property	Typical range	Beatson 1H set (1062 cpds)		
		Minimum	Maximum	Mean/median
Heavy atom count	8–20	8	23	14
Polar surface area	≤110	3.2	120.9	47.1
Hydrogen bond donors	≤3	0	3	1
Hydrogen bond acceptors	≤4	1	7	3
Ring count	≤4	0	4	2
cLogD (pH 7.4)	−3 to 3	−3.4	3.6	0.5
Rotatable bond count	≤4	0	6	2

Similarity screening against already chosen fragments, examining 2D fingerprints and/or 3D similarity, facilitates library diversity. Unique hits are more likely to be identified through the inclusion of a diverse set of fragment shapes containing enthalpy‐driven pharmacophores, which would increase the sampling efficiency of the relevant chemical space. Furthermore, the inherent chemical stability and reactivity must also be considered, along with the exclusion of any toxicophoric liabilities. To this end, regular quality control (QC) of fragment libraries is important to ensure only high‐quality compounds are screened. Pan‐assay interference compound (PAINS) filters can be used to remove molecules, which bind nonspecifically to numerous biological targets. Several frequent hitters with little potential for advancement have also been identified and should be avoided for this reason. As discussed in detail below, several computational methods can be used both for property prediction and filtering purposes.

There have been several discussions in recent years regarding the inclusion of a higher degree of 3D fragments within screening libraries [24], with some raising concerns that this would lead to a lower hit rate. However, hit rate does not define the success of a library as it is more important to identify ligand‐efficient and chemically tractable start points. Increasing the percentage of 3‐dimensionality (or Fsp3) has the potential to cover a broader range of biologically relevant chemical space, improving the potential medicinal chemistry start point [23, 25, 26], with ‘frequent hitters’ (compounds which appear as actives in many unrelated screens and which may, therefore, lack specificity) generally falling within the low Fsp3 range. It has been shown that increasing sp3 character may improve several compound properties and contribute to clinical success. In particular, incorporation of out‐of‐plane functional groups within a 3D structure can potentially enable stronger receptor/ligand interactions, thus improving potency and selectivity to a given target [26].

**Fig. 3:**
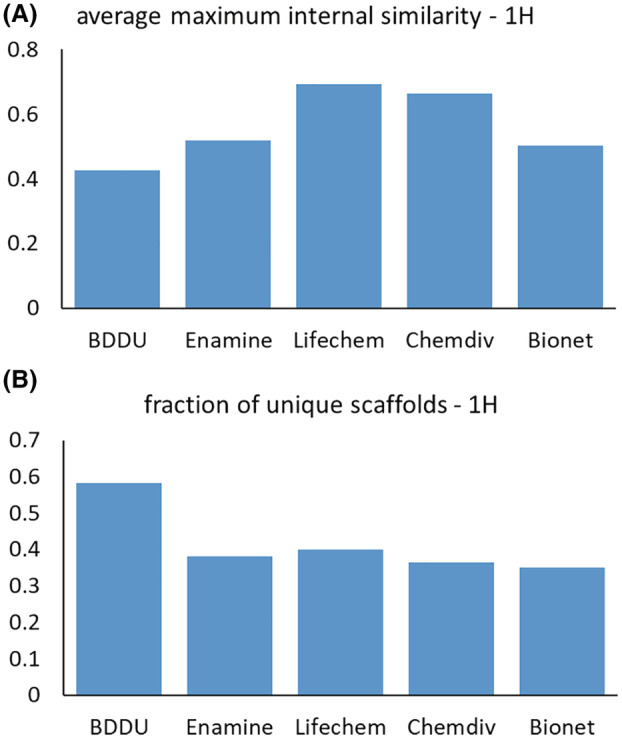
Comparison of Cancer Research UK Beatson Drug Discovery Unit 1H fragment set against selected commercial sets. (A) Average maximum internal similarity. For each compound in any set, Tanimoto similarity was calculated against all other compounds in the set using Morgan fingerprints (radius 2) in RDKit. The maximum value was retained for each compound and averaged over the set. (B) Fraction of unique Bemis–Murcko scaffolds [29] in each set. Scaffold SMILES were extracted for each compound in each set using vortex software (Dotmatics, Bishops Stortford, UK) and unique canonical SMILES retained. The number of unique scaffolds was expressed relative to number of compounds in each library. Commercial sets: Enamine. High Fidelity Library (1920 compounds); Life Chemicals, General Fragment Library (50 607 compounds); ChemDiv, Fragments Library (11 269 compounds); Bionet, 2nd Generation Premium Library (1166 compounds). SMILES, simplified molecular‐input line‐entry system. [Colour figure can be viewed at wileyonlinelibrary.com]

Does library size matter? Yes and no. The majority of successful FBDD campaigns utilise libraries ranging from 1000 to 2000 compounds [27]; however, the diversity of the library is more important than the overall number. A study conducted by von Itzstein showed that only ~ 2000 fragments are required to represent the same level of true diversity as an overall set of > 220 000 [27]. Therefore, playing the numbers game is not necessary, but instead it is more beneficial and cost‐effective to design a smaller library with a high degree of diversity (Fig. 3). Recently, small libraries such as ‘SpotXplorer’ [28] have been designed to maximise the coverage of experimentally confirmed binding pharmacophores at protein hotspots. The efficiency of this approach was demonstrated with a library of only 96 fragments that were validated on popular target classes, such as G‐protein coupled receptors (GPCRs), as well as emerging targets such as Su(var)3‐9, Enhancer‐of‐zeste and Trithorax‐Domain containing 2 (SETD2).

#### 2.2.2 Computational library design

One approach to fragment library design is to start from known bioactive molecules. Thus, fragments can be obtained from deconstruction of larger molecules according to some ‘breaking rules’ and commercial availability determined for promising candidates. Fragments which make contributions to binding known targets can be determined, for example, by searching in BindingDB [30]. The most well‐known methods to decompose existing molecules are RECAP (REtrosynthetic Combinatorial Analysis Procedure) and BRICS (Breaking of Retrosynthetically Interesting Chemical Substructures) [31, 32].

RECAP identifies fragments in existing molecules by breaking bonds generated by common chemical reactions. The cleavage involves only 11 chemical bond types, and all the bonds are broken in a single step. Ring motifs are left intact. Since its very early stages, RECAP developers allowed user selection of alternative bond types and the code was subject to several modifications over the years [32, 33, 34]. Among RECAP modifications, BRICS is one of the most popular and involves the inclusion of a complementary set of rules for the recombination of the chemical space (such as modelling of ring substitution and cleavage of sulfur groups), leading to the definition of 16 fragment prototypes [31]. It has been shown that these modifications generally lead to the generation of a larger number of fragments with a smaller size than the ones obtained using RECAP rules [35]. Additionally, more fragments with greater than one connection point resulted from BRICS [31], which means more branching possibilities in the obtained subset.

However, since a key advantage of FBDD is its efficient sampling of chemical space, a library based solely on available fragments from known bioactive compounds would likely not be desirable and augmentation would be required. As an example, Selcia Ltd developed their commercial library of 1366 fragments through an initial selection based on curation of RECAP‐generated bioactive fragments meeting Ro3 and a calculated solubility threshold. These were supplemented with under‐represented fragment classes and by a custom synthesis programme targeting noncommercially available fragments to improve novelty (https://www.selcia.com/sites/default/files/SelciaFragmentLibrary.pdf).

Commercial fragment sets are often broadly Ro3 compliant (Fig. 2) [36]. However, specific types of fragments may also have unique property distributions, as discussed below, and further property filtering can be performed according to evolving needs in order to augment the background composition of the library. Importantly, one should not assume the absence of unwanted functionality in commercial sets, especially with larger collections. Therefore, sub‐structural searches are performed to identify liabilities (see [37] and references therein), typically using filters expressed in Daylight SMARTS format (SMILES arbitrary target specification format, https://www.daylight.com/dayhtml/doc/theory/theory.smarts.html, in which SMILES refers to the string‐based simplified molecular‐input line‐entry system molecular representation [38]).

Maintaining diversity is also critical. A simple first step is similarity screening utilising appropriate cut‐offs in Tanimoto similarity of 2D fingerprints to exclude compounds in a vendor set that are highly similar to existing library fragments. A detailed discussion on the most used molecular fingerprints goes beyond the scope of this review, and interested readers are referred to [39]. For a pair of binary fingerprints, Tanimoto similarity is the ratio of the sizes of the sets comprising the intersection/union of on‐bits in each fingerprint. Therefore, if all elements are shared in both fingerprints, similarity is one. If no elements are shared, similarity is zero. To measure the impact of potential compound additions to a library, analysis of intra‐set similarity of the library with or without the new candidate compounds can be performed. Filtering can also be done with pharmacophore fingerprints. A pharmacophore is defined as the optimal steric and electronic features necessary to ensure the optimal ligand/receptor interactions [40]. Pharmacophore modelling represents molecules as collections of features at the 2D or 3D level [41]. This information is binary encoded to pharmacophore fingerprints, indicating the presence or absence of pharmacophore features together with ligand topological information. Pharmacophore fingerprints are thus particularly useful to analyse similarity and remove redundancy [28, 41].

The need for optimal molecular complexity is a foundational concept in FBDD [18], and various metrics of synthetic tractability and structural complexity have been developed which can be useful in filtering fragments [42, 43, 44]. Improving shape diversity of libraries has also received increasing attention in recent years [24]. Where 3D character of the library is of particular interest, analyses that are less computationally expensive than 3D pharmacophore shape similarity can also be performed. Fraction of sp3‐hybridised carbons (Fsp3) is a simple calculated property which, as noted above, has been associated with improved pharmacokinetic properties and clinical success [26, 45]. Principal moments of inertia (PMI) express the torque required to cause a change in angular acceleration of a rigid body (a molecule, in this case) around orthogonal axes of rotation. When appropriately normalised, triangular PMI plots indicate the extent to which a molecule is rod‐like, disc‐like or sphere‐like [46]. Plane of best fit (PBF) is another 3D shape metric, which calculates average distances of all heavy atoms in a single calculated conformer away from a best‐fit plane minimising this average [47].

An interesting approach for quantitative structure–activity or structure–property relation modelling (QSAR/QSPR) utilising chemical graph theory combined with SMILES notation was recently reported [48]. In this method, a graph (a set of nodes, representing atoms, and edges representing bonds) is built using the connectivity information of an input molecule and molecular fragments are obtained from the possible subgraphs. All unique fragments obtained in a data set of structures can be collated, and counts of each fragment in individual molecules can then be used as descriptors in QSAR/QSPR models. Interestingly, fragments associated with activity in trained models could be retrieved [48] suggesting this could also be used as another plausible approach to fragment selection, though we are unaware of an example of this use in library construction. The Reymond group also previously reported the ‘chemical universe’ database GDB‐17, comprising enumeration of all chemical graphs consisting of C, N, O, S and halogens up to 17 heavy atoms [49]. Subsequently, the same group released a low‐complexity subset of 10 million of these for use in virtual screening such as QSAR approaches [50].

Beyond standard techniques to filter commercial sets for the selection of new fragments, new approaches to *in silico de novo* molecular design driven by advances in artificial intelligence could have applications in library generation through automated design of novel fragments with optimal properties of interest [51]. *De novo* design refers to virtual generation of novel compounds fulfilling criteria such as likely target binding and has been investigated for decades [52, 53, 54]. *De novo* design approaches are broadly categorised as receptor‐based (where the structure of a target binding site is known) or ligand‐based (for example, using 3D pharmacophores of known binders without any protein structure information) [55]. Recently, considerable effort in this area has been focussed on generative neural networks, which are trained to produce novel molecules by learning features of large and diverse compound sets. The reader is referred to [51, 56] for in‐depth reviews of this area. Briefly, the majority of generative chemistry frameworks reported to date are broadly based on autoencoders (AE), generative adversarial networks (GAN) or, more recently, transformer models.

Autoencoder consist of encoder and decoder parts. The encoder produces a dimensionally reduced ‘latent variable’ representation of its input. The decoder receives this as input and learns to reconstruct each input training example at the output [57]. Variational autoencoders (VAEs) are similarly structured, although in this case the training objective includes a term which forces the latent variable distribution to be close to a desired preselected prior distribution (typically Gaussian). This addition enforces regularisation on the learned latent space [58].

In contrast, GAN consists of generator and discriminator networks. The generator draws random samples from a multivariate prior distribution and transforms these into candidate examples of the data of interest. The discriminator scores examples presented and attempts to classify them as real or fake. Both models are simultaneously trained in adversity, resulting in a 2‐player zero‐sum game in which improvement of one model leads to decreased performance of the other [59]. Thus, improvement in the generator corresponds to the production of samples that more closely match the distribution of real data, as perceived by the discriminator, by sampling from random noise.

For both model types, by sampling and decoding from the learned latent representations or learned distributions, novel molecules not seen in training can be generated. Frequently, such models have been trained to directly output the SMILES representation of novel molecules [38]. In AE/VAE frameworks, this is a ‘sequence to sequence’ learning task suited to recurrent neural networks (RNN) [60]. However, effective learning of long‐range dependency and context in longer sequences can be problematic for RNN. Improvements can be made by the introduction of ‘attention’ mechanisms, which encode information on positional context [61]. Transformers extend this concept by using an ‘attention‐only’ framework that eliminates the need for RNN in sequence‐based tasks [62]. Recently, this newer approach has also been investigated for molecular optimisation and reaction prediction [63].

A range of other molecular representations are also utilised in addition to SMILES. Deep‐SMILES and self‐referencing embedded strings (SELFIES) are alternative string representations developed specifically for generative modelling and which address the problem that syntactically incorrect (invalid) strings are often returned by SMILES‐based generators [64, 65]. Interestingly, a fast generative algorithm which eliminates the need for machine learning models was recently reported using SELFIES [66]. Generative models using molecular graphs have also been reported (reviewed in [67]). Voxel‐based representations can be used for 3D generation [68]. Another approach to 3D generation used a wave transform to overcome sparsity of voxel representations [69].

Most applications of *de novo* molecule design in drug discovery are naturally targeted at producing drug‐like molecules, although the model frameworks above are equally suited to fragment generation. We recently reported a fragment autoencoder model trained to reproduce both SMILES and chemical fingerprints [43]. Using in‐house data from previous screens, we applied transfer learning to the fingerprint decoder layers to develop a model that scores the likelihood that novel generated molecules will be ‘privileged’ fragments (capable of binding to multiple protein targets [70]). Our sampling approach used particle swarm optimisation [71] to simultaneously optimise for privileged fragment scores, synthetic accessibility and Fsp3, among other criteria. A similar sampling approach was also reported by Winter et al. [72] to identify potential Epidermal Growth Factor Receptor (EGFR) and β‐site amyloid precursor protein cleaving enzyme 1 (BACE1) inhibitors while simultaneously optimising against support vector models of several absorption, distribution, metabolism, excretion and toxicity (ADMET) properties.

In another fragment‐based approach, Arus‐Pous et al. [73] developed a ‘scaffold decorator’ model. This consists of a scaffold generator model, which outputs fragments with defined attachment points. These are subsequently modified by a decorator model which adds Ro3‐compliant groups to each attachment point. In one experiment, the authors trained the model using a set of scaffolds and decorations obtained by fragmenting known dopamine receptor D2 (DRD2) modulators. The model was then able to generate novel molecules with *in silico* predicted activity when diverse new scaffolds were used. Such an approach could potentially be utilised to suggest fragment hit growth strategies against a given target. We further discuss potential applications of generative modelling in fragment elaboration below.

### 2.3 Different types of fragment libraries and some considerations

#### 2.3.1 19F

NMR is both the oldest and most robust technique used for the detection of weak binders [74], with Shuker et al. having originally reported ‘SAR by NMR’ in 1996 [75]. Since then, the field has grown substantially, and heteronuclear spectroscopy methods (which detect chemical shifts originating from nuclei other than 1H, such as 19F) are now widely used alongside 1H NMR spectroscopy for the identification of novel binders. With that in mind, the design of fragment libraries containing fluorine atoms for 19F NMR screening is now a key component in the fragment screening process. The general library design considerations outlined above should be applied to 19F fragment libraries, with the obvious caveat that molecules must contain at least one fluorine atom.

1H screening relies on fragment cocktails, which need to be carefully designed to limit signal overlap. In contrast, the use of 19F‐containing fragments enables simpler analysis of spectra due to the wider chemical shift dispersions and minimal overlap with background signals. As a result, 19F fragment libraries can be screened in cocktails of approximately 20 compounds, while standard cocktails contain only 5–6 molecular entities [76]. Interestingly, it has been shown that a library size of ~ 1200 fluorinated compounds can achieve similar levels of diversity to a set of ~ 2000 standard fragments [27]. Inclusion of fluorine can be an added advantage due to the improved physicochemical and metabolic properties, which are associated with using it as a bioisostere. Thus, its removal is not required during elaboration if it enhances interactions and/or improves ADME properties of lead compounds.

#### 2.3.2 Covalents

Although standard 1H and 19F NMR libraries account for the majority of FBDD screens, a number of newer technologies have recently come to fruition. With the resurgence in interest towards covalent inhibitors, the field of covalent fragments has garnered attention [77, 78, 79, 80]. All covalent fragments contain a reactive electrophilic functional group, generally capable of forming an irreversible bond with an amino acid residue. In addition to standard FBDD considerations, the stability (both inherent and to physiological conditions), reactivity and size of the electrophilic functionality must also be taken into account when designing covalent fragments. Unlike traditional fragment screens, desirable parameters may change depending on the targeted protein.

Library design may, therefore, be influenced by the nature of the amino acid residue [81] and its location within the active site [82]. The nucleophilicity and pKa [83] of amino acid side chains can vary depending on the protein environment, and thus, a less reactive amino acid residue may require a more reactive warhead for efficient reaction. It is, therefore, desirable to maintain a library containing a range of reactivities [84], as well as varying electrophilic functional groups [85]. It is worth noting that the incorporation of highly reactive warheads within a screen may lead to the identification of lower affinity binders, with the inactivation rate constant (*k*_inact_) playing a more significant role in the binding event, due to covalent bond formation, than the inhibition constant (*k*_i_) resulting from reversible binding.

As well as considering the reactivity of a warhead, it is optimal for the electrophilic functionality to be appended by a minimal linker and not embedded within the fragment scaffold. This is largely because the geometry of the warhead and angle of attack have a significant role in the formation of the desired covalent bond and, thus, ease of access to the warhead is more likely to allow hit identification. Covalent hits can be grown and merged using traditional fragment strategies [86] to enhance the binding affinity through noncovalent interactions. It may even be possible to remove the warhead and maintain affinity once the scaffold is optimised. To this end, a covalent approach may be favoured to aid in the identification of lower affinity allosteric sites. However, this approach is only amenable when a suitable nucleophilic residue is present. Caution should also be taken to ensure binding occurs within a ‘real’ site, as with any fragment hit, and is not a result of elevated fragment electrophilicity.

Screening of covalent fragments can be carried out by NMR as with traditional fragment sets. In fact, peaks are often more pronounced with visibly increased chemical shift perturbation in multi‐dimensional heteronuclear experiments, allowing for easier analysis. Screens for high‐profile targets such as bromodomain‐containing protein 4 (BRD4) [87] and KRas [88] have been carried out in this way. Despite this, NMR is generally underutilised and screening via simpler MS studies is often employed instead [89]. Liquid chromatography with tandem mass spectrometry (LC–MS/MS) allows accurate detection of whether covalent binding has taken place in a high‐throughput manner. Native MS is often combined with time‐of‐flight (TOF) instruments to enable high sensitivity detection of both the target and fragments [77]. A digestion protocol may also be utilised to determine exactly which amino acid has reacted. Covalent fragment libraries of 100–1400 compounds (predominantly acrylamides and chloroacetamides) have been screened in this way to identify binders for well‐known targets such as Janus Kinase (JAK) [90] and KRas [89].

Covalent fragment docking algorithms were recently introduced as an *in silico* approach to discover reversible and irreversible fragment inhibitors [91, 92]. Screens using other assay types have also been reported. A nucleotide exchange assay was utilised to identify KRAS^G12C^ mutant binders via Carmot Therapeutics *Chemotype Evolution* technology, entailing rapid synthesis and testing of libraries based on an existing fragment‐like molecule [93]. This generated a custom library of ‘beyond rule of 3’ fragments through pharmacophore linking. The acrylamide compounds were not purified before screening and ultimately led to the discovery of AMG‐510 (sotorasib), which was granted FDA approval for the treatment of nonsmall cell lung cancer (NSCLC) in 2021 having only entered the clinic in 2018. Notably, it took only 8 years from the initial publication by the Shokat group in 2013 [89], demonstrating the druggability of the KRAS^G12C^ mutant, to treating real‐life patients.

#### 2.3.3 Fraglites and mini frags

In 2019, Waring et al. [94] and Jhoti et al. [95] independently reported the use of ‘Fraglites’ and ‘Minifrags’ for the identification of ligand–protein interactions. Waring et al. hypothesised that sites of interaction could be identified using a small library of molecules with minimal molecular weight (≤ 13 heavy atoms) and complexity. Therefore, they utilised a set of compounds containing a ‘pharmacophore doublet’ capable of forming two polar bonds but with different spatial orientations. Halogens were included alongside these paired hydrogen‐bonding motifs to allow unambiguous identification in X‐ray crystallography, utilising the unusual scattering of the halogen substituent. A set of 31 ‘FragLites’ were selected to encompass all combinations of pharmacophore doublets with a high degree of aqueous solubility for X‐Ray crystallography screening. The utility of the approach was demonstrated through mapping of cyclin‐dependent kinase 2 (CDK2), identifying both orthosteric and allosteric sites, with hits being quickly developed into lead‐like molecules [94].

Similarly, the ‘Minifrags’ approach from Astex also utilises highly soluble, ultra‐low molecular weight compounds (average HAC < 7), designed to sample chemical space [95]. A minimal set of 81 compounds allowed the identification of hot and warm ligand‐binding spots for potential targeting on proteins, such as ERK2. The Minifrag set was found to have both a higher hit rate and to identify a larger number of theoretically druggable sites, than a more conventional X‐Ray set of 440 compounds. These approaches may hold advantages in the future, allowing the identification of new target sites with a minimal compound screen. A version of the MiniFrag screening set has already been used in the identification of hits against SARS‐CoV‐2 main protease [96].

#### 2.3.4 Phabits

Recently, the field of FBDD has expanded to include photoaffinity‐based screening approaches, with Bush et al. reporting the use of ‘Phabits’ for the identification of protein–ligand interactions through covalent capture [97]. The methodology utilises photoreactive fragments which, upon irradiation with light, crosslink to proximal protein residues in a biochemical setting. Hits can then be identified by intact protein LC–MS, with follow‐up studies to determine binding affinity and the site of crosslinking. This follows earlier work reported by Cravatt and co‐workers where photoreactive fragments were used for the identification of fragment–protein interactions in live cells [98]. Phabits utilises purified protein to enable high‐throughput and targeted screening against proteins of interest, which was demonstrated in the paper through the identification of binders to KRAS^G12D^ and BRD4‐Protacs using a mere 556 fragments. Identified hits can immediately be used as reporters in displacement assays to screen for more potent binders in a site‐specific manner. Despite potential future advantages, access to commercially available photoreactive fragments is still poor. In addition, some target classes, such as membrane‐bound proteins, are unlikely to be responsive to the approach, as they often need to be stabilised in a lipid bilayer. Moreover, crosslinking yields are often low and do not always correlate with affinity [99].

## 3 Growing a fragment

As with any screening campaign, hits need to be prioritised to focus resources. But what makes a good fragment hit [100]? Consideration of multiple parameters is necessary. Biological activity is obviously one of the most important, and so target binding validation and generation of parameters such as ligand efficiency (LE) or lipophilic efficiency (LiPE) can help facilitate proper comparison [15]. As a generalisation, growing a molecule will add lipophilicity and so a more hydrophilic hit may be advantageous. In addition to this, it is important to consider a number of other factors: solubility, the availability of commercial analogues and starting materials, overall synthetic tractability and, perhaps most importantly, the availability of binding mode structural information. The availability of close analogues for validation and immediate SAR is highly important as it will determine how rapidly a project can be progressed [101]. Frequent hitters and unwanted functionality should be discounted at this point. Although, with a properly designed screening library hits of this type should be minimal.

Growing the hit to increase the size of the molecule and include additional functionality is the most straightforward approach to go from a fragment to a drug‐like molecule. Identification of growth vectors and potential points of interaction with the target is important for rational design and can be difficult without the aid of a crystal structure. To this end, X‐Ray crystallography has become an increasingly popular screening method for rapid hit exploration, with platforms such as XChem (https://www.diamond.ac.uk/Instruments/Mx/Fragment‐Screening.html [96]) and FragMAX [102] now widely available. Several groups have also explored the screening of crude reaction mixtures via this method [103, 104]. However, growing crystals can be challenging, resolution can be poor [105], and secondary techniques are still required to determine binding affinities.

In cases where structural information is unavailable, evidence can be gained from NMR experiments or fragment/receptor complex obtained from docking calculations can be used as an educated guess [106]. Docking calculations allow predicting receptor/ligand‐binding motifs and assigning a ranking score to the obtained binding poses. In the most fortunate cases, the docking score can be directly correlated with the experimental binding affinity. Assessment of the results, using existing receptor/ligand crystallographic data with known experimental binding affinities, is always a good practise, especially for cases where the receptor shows high flexibility.

Docking can be applied both in screening a fragment library and to assist in fragment elaboration. Usually, docking is performed with a flexible ligand and a rigid receptor, treating the fragment core(s) as fixed. However, most of the time this assumption is not true, because receptor conformational changes occur upon binding. Therefore, techniques such as induced‐fit docking [107] and molecular dynamics (MD) are also used to assess the predicted binding motifs by docking. Due to the higher computational cost of the method, induced fit is usually kept for refinement purposes and not used at the very early screening stages. A faster and cheaper way to consider protein conformational freedom when screening a library of the order of thousands of compounds is to perform rigid receptor docking calculations on different receptor conformations, coming either from experimental data or obtained beforehand making use of MD simulations. These can be coupled with enhanced sampling techniques such as accelerated MD [108] and metadynamics [109] in order to speed up the apo‐receptor space exploration and assign the protein conformations a converged probability estimation. This can be treated as a conformational receptor probability score and used to average and reweigh the docking scores [110]. In this context, apo‐receptor simulations can be extremely useful when the receptor structure is not crystallised and is constructed via homology modelling or obtained from an AlphaFold prediction [110, 111].

A common issue to most docking calculations is that typical scoring functions are not able to accurately predict poses far from the known bioactive ligand. Binding poses can always be improved by enhancing the exploration of the ligand conformational space. This can be done using molecular dynamics simulations, for example, or enhanced sampling techniques such as metadynamics [112, 113]. These algorithms are computationally more expensive and, therefore, not advised to be used for the initial screening phase, but for a refinement stage on a selected fragment subset. Docking scores can also be complemented by a more accurate estimation of the binding affinity, using molecular mechanics Poisson–Boltzmann surface area (MM/PBSA) [114], which provide a reasonable trade‐off between speed and accuracy [115].

Although docking is an established technique for HTS, it has only recently started to be systematically employed for fragment libraries. The small size of fragments together with their weak affinity and dynamic binding motifs make computational structure‐based fragment virtual screens challenging. Moreover, the absence of a complete data set of protein–fragment complexes complicates validation and docking results assessment. Nevertheless, several studies have shown acceptable performance of the most used docking programmes for small molecules [116, 117, 118, 119, 120].

Once structural information of known ligand–target complexes is known, techniques such as scaffold hopping can be used to substitute a central element of the molecular scaffold by a new molecular fragment [121]. In an ideal scenario, the features of both initial building blocks should additively contribute to affinity. However, geometry is key and so several linking/merging options might need to be considered [122]. From the computational perspective, several techniques can be used to estimate binding affinity. Among these, we name MM/PBSA [115] and free energy perturbation (FEP) [123], the latter proven to be particularly effective for ligand optimisation, specifically when small changes in the ligand design are introduced.

The recent explosion in machine learning‐based *de novo* design methods also provides numerous approaches with the potential to assist in fragment elaboration. Besides the scaffold decorator model discussed earlier [73], Lim et al. [124] trained a graph‐based VAE using dual inputs of molecules and their Bemis–Murcko scaffolds [29]. The model could then generate new molecular graphs by sequentially adding atoms and bonds to a provided scaffold. Additionally, generation could be conditioned on molecular properties. Green et al. also recently reported a convolutional neural network trained to predict a unique fingerprint corresponding to a fragment that could be added in a known receptor/ligand structure to improve binding affinity of the known input ‘parent’ ligand [125, 126]. Predicted fingerprints could then be matched against a fingerprint library of known fragments.

Olivecrona et al. trained a recursive neural network SMILES generator using reinforcement learning (RL) and illustrated its use on several tasks including similarity‐ and target activity‐guided generation [127]. RL combines a generator with a ‘critic’ which assigns reward to generator outputs. The generator is trained to maximise this expected reward. The target‐activity task required a training data set of active/inactive compounds against the chosen target (DRD2 receptor), which would likely be lacking in early hit elaboration for novel targets. However, RL can also be used for property‐guided generation [128, 129]. Stahl et al. used an explicit fragment‐based encoding of molecules in their RL model [129].

Another approach which could be applied to generate molecules which are similarly structured to a fragment hit but with properties in a target range is mol‐cycleGAN [130]. The cycleGAN method [131] aims to provide a mapping between two unpaired data domains, X and Y (one example from its original use in image translation is photographs of horses and zebras which are not directly paired). This consists of two coupled GAN models. One model aims to learn to translate elements of X to resemble elements of Y (horse → zebra, for example). The other model aims to learn the inverse mapping. The models are trained together with a ‘cycle‐consistent’ objective such that an element of X translated into the domain Y by the first network should map back to itself through the second GAN. In use, one network is used. For example, an image of a horse may be given zebra‐like stripes [131]. In mol‐cycleGAN, the training sets could be inactive/active compounds or sets which diverge in another property of interest. The method was used for several tasks including optimising cLogP while retaining structural similarity, in addition to a predicted DRD2 activity optimisation task [130].

The aforementioned studies are a small sample of a rapidly growing field, and a thorough review is beyond this work. However, we note that the excitement surrounding novel AI‐driven *de novo* methods in drug discovery derives from the suggestion that these approaches could be used to arrive more or less directly at the clinical candidate (or at least drastically reduce the time spent in design‐make‐test‐analyse cycles). To date, one of the most successful companies in this space, Exscientia, and its partners have progressed three molecules discovered in accelerated programmes with the use of its design platform into phase I [DSP‐0038, a dual 5‐hydroxytryptamine (5‐HT) 1A/2A antagonist; EXS‐21546, an adenosine A2A receptor antagonist; and DSP‐1181, a 5‐HT1A antagonist] (https://www.exscientia.ai/). In this context, one could ask whether *de novo* design might supersede FBDD. However, many publications which have applied AI‐based design to specific targets have focussed on well‐known and previously drugged targets for which relatively large bioactivity data sets are available, such as DRD2. Therefore, the impact that AI‐based *de novo* design will have on very difficult targets, the area in which FBDD excels, remains to be seen. Nevertheless, this is a rapidly developing field and strategies that integrate structure‐based information to drive improvements in generation are of particular interest [132].

## 4 Conclusion

In this review, we have aimed to give the reader an appreciation of key considerations in designing a fragment library, in addition to an overview of emerging technologies, both chemical and computational, which are likely to accelerate FBDD. As we noted at the start, the use of an FBDD approach has resulted in six marketed drugs to date and many additional clinical candidates. Although many of these agents were discovered using ‘classical’ FBDD approaches, the impact of newer FBDD technologies is already being felt by patients. We noted above the rapid development of Sotorasib, which was granted FDA approval in 2021 only 8 years after the initial demonstration of the druggability of the KRAS^G12C^ mutant. By comparison of asciminib, the most recently approved drug discovered through a more traditional FBDD approach entered clinical trials in 2014. This is even more impressive when one considers that KRAS was, until this point, considered ‘undruggable’. We believe this example illustrates how an emerging arsenal of new FBDD technologies and intelligent library design may finally lead to progress against some of the most difficult targets in drug discovery that have proven intractable until now.

## Conflict of interest

The authors declare no conflict of interest.

## Author contributions

MB, AB and KM involved in visualisation, writing−initial draft and writing−review and editing; JB involved in conceptualisation, funding acquisition, resources, supervision, writing−initial draft and writing−review and editing; MB, AB and KM contributed equally to this work.

### Data availability statement

The commercial datasets used and/or analysed during the current study are available from the corresponding author on reasonable request. However, the commercial fragment libraries analysed are available by registration on the websites of the respective vendors. Specific composition of the Beatson 1H fragment set is proprietary.
